# Development of a serious game for learning about safe sex and contraception in adolescence ^*^

**DOI:** 10.1590/1518-8345.7036.4182

**Published:** 2024-06-17

**Authors:** Lilian Mayumi Chinen Tamashiro, Luciana Mara Monti Fonseca

**Affiliations:** 1 Universidade de São Paulo, Escola de Enfermagem de Ribeirão Preto, PAHO/WHO Collaborating Centre for Nursing Research Development, Ribeirão Preto, SP, Brazil.; 2 Scholarship holder at the Conselho Nacional de Desenvolvimento Científico e Tecnológico (CNPq), Brazil.

**Keywords:** Adolescent Health, Sexuality, Health Education, Contraception, Mobile Applications, Technology, Salud del Adolescente, Sexualidad, Educación em Salud, Anticoncepción, Aplicaciones Móviles, Tecnología, Saúde do Adolescente, Sexualidade, Educação em Saúde, Anticoncepção, Aplicativos Móveis, Tecnologia

## Abstract

**Objectives:**

to develop a serious game in a mobile application for learning about safe sex and contraception with the active participation of adolescents; and evaluate the content, appearance and usability of the technology with adolescents and experts.

**Method:**

this is an applied research project into the development of a serious game, carried out in two stages: development of the technology itself; and evaluation of the content, appearance and usability of the application. Teenagers from a public school in the city of São Paulo-Brazil took part in the technology development stage. The evaluation was carried out by the adolescents and experts in the areas of public health and health technology. The following criteria were used: educational aspects, environment interface and didactic resources.

**Results:**

Prinventon App ^®^ was developed, a serious game set in a virtual city, designed to address adolescent sexuality. The app received 90% positive responses and had a Content Validity Index of 0.80, which was considered adequate. The suggestions and notes were accepted and implemented. The serious game was considered interesting and important in terms of the subject matter.

**Conclusion:**

it was found that the technology developed can help adolescents learn about safe sex and contraception, by addressing sexuality in adolescence in a playful and realistic way.

## Abstract (es)

**Objetivos:**

desarrollar un *serious game* en aplicación móvil para el aprendizaje sobre sexo seguro y anticoncepción con la participación activa de los adolescentes; y evaluar contenido, apariencia y usabilidad de la tecnología con adolescentes y expertos.

**Método:**

se trata de una investigación aplicada, de producción tecnológica acerca del desarrollo de un *serious game* , realizada en dos etapas: desarrollo de la tecnología propiamente dicha; y evaluación del contenido, apariencia y usabilidad de la aplicación. Adolescentes de una escuela pública de la ciudad de São Paulo-Brasil participaron en la etapa de desarrollo de la tecnología. Ya la evaluación fue hecha por los adolescentes y expertos en las áreas de salud colectiva y tecnología en salud. Se siguieron los criterios: aspectos educacionales, interfaz del ambiente y recursos didácticos.

**Resultados:**

se desarrolló *Prinventon App* ^®^ , un *serious game* ambientado en una ciudad virtual, diseñado para abordar la sexualidad en la adolescencia. La aplicación obtuvo 90% de respuestas positivas y presentó Índice de Validez de Contenido de 0,80, siendo considerado adecuado. Las sugerencias y apuntes fueron acogidos e implementados. El *serious game* fue considerado interesante e importante ante el tema abordado.

**Conclusión:**

la tecnología desarrollada puede ayudar a los adolescentes en el aprendizaje sobre sexo seguro y anticoncepción, al apuntar abordar, de manera lúdica y realista, la sexualidad en la adolescencia.

## Abstract (pt)

**Objetivos:**

desenvolver um *serious* *game* em aplicativo *mobile* para aprendizagem sobre sexo seguro e contracepção com a participação ativa dos adolescentes; e avaliar conteúdo, aparência e usabilidade da tecnologia com adolescentes e *experts* .

**Método:**

trata-se de uma pesquisa aplicada, de produção tecnológica sobre o desenvolvimento de um *serious* *game* , realizada em duas etapas: desenvolvimento da tecnologia propriamente dita; e avaliação do conteúdo, aparência e usabilidade do aplicativo. Adolescentes de uma escola pública da cidade de São Paulo-Brasil participaram da etapa de desenvolvimento da tecnologia *.* A avaliação foi realizada pelos adolescentes e *experts* nas áreas de saúde coletiva e tecnologia em saúde. Seguiram-se os critérios: aspectos educacionais, interface do ambiente e recursos didáticos.

**Resultados:**

desenvolveu-se *Prinventon App* ^®^ , um *serious* *game* ambientado em uma cidade virtual, projetado para abordar a sexualidade na adolescência. O aplicativo obteve 90% de respostas positivas e apresentou Índice de Validade de Conteúdo de 0,80, sendo considerado adequado. As sugestões e apontamentos foram acatados e implementados. O *serious* *game* foi considerado interessante e importante diante do tema abordado.

**Conclusão:**

a tecnologia desenvolvida pode auxiliar os adolescentes na aprendizagem sobre sexo seguro e contracepção, ao abordar, de maneira lúdica e realística, a sexualidade na adolescência.

## Introduction

 In 2020, it was estimated that approximately 133.8 million Brazilians used the internet in their daily lives ^(^ 1 ^)^ . The smartphone was the most popular device, adopted by 99% of users, surpassing other devices. Among its biggest enthusiasts are teenagers, with around 10 million of them worldwide accessing the internet on a daily basis. Their main online activities include interacting on social networks, entertainment, cultural enrichment, learning and searching for information ^(^ 2 ^)^ . 

 Adolescence plays a significant role in the sphere of public health, as it is during this phase that sexual practices generally begin, placing adolescents in a context of vulnerability with regard to sexually transmitted infections (STIs), unintended pregnancy and abortion ^(^ 3 ^)^ . The World Health Organization (WHO) ^(^ 4 ^)^ defines adolescence as the period between the ages of 10 and 19, when teenagers begin to seek greater autonomy. This situation creates an ambivalence, since, on the one hand, they are not expected to take on all the responsibilities of adult life, but, on the other hand, they are not given the space to act like children ^(^ 5 ^)^ . 

 According to the National School Health Survey - PeNSE, conducted in 2019, 35.4% of adolescents aged between 13 and 17 have already started having sex. Among them, only 59.1% used a condom during their last sexual intercourse, and 7.9% of adolescents have become pregnant at least once ^(^ 6 ^)^ . In addition, in 2021, Brazil recorded around 19,000 births per year to mothers aged between 10 and 14, resulting in a rate of 53 pregnant teenagers per thousand, surpassing the global average of 41 cases, indicating that the teenage pregnancy rate in Brazil is above the world average ^(^ 7 ^)^ . 

 As for HIV (Human Immunodeficiency Virus)/AIDS (Acquired Immunodeficiency Syndrome) cases, between 2010 and 2021, there were a total of 32,256 reported cases of adolescents aged between 10 and 19 living with these conditions, with 1,156 new cases reported in 2021 alone ^(^ 8 ^)^ . 

 According to data provided by the São Paulo State Health Department, there was a reduction in the incidence of teenage pregnancy in the state in 2022. This group of adolescents aged 15 to 19 comprised 41,595 pregnant women, equivalent to 8.1% of all births in the state of São Paulo ^(^ 9 ^)^ . 

 In this context, sex education is of great importance in guiding the conduct and decisions of adolescents in relation to their sexual and reproductive health, with the aim of preventing potential risks, and is thus an important tool capable of bringing about significant changes in the behavior of a population ^(^ 5 ^)^ . 

 Currently, with the growing development of technologies, especially those aimed at health, the so-called eHealth or digital health, the possibilities for personalized intervention, implementing health promotion actions and influencing behavioral changes related to care are also increasing ^(^ 10 ^)^ . 

 With this in mind, we can cite the smartphone as a technological tool to support the learning process aimed at sex education. In this sense, “serious games” play a crucial role, as they are electronic games with specific purposes and content for education, designed to motivate the learning process, widely used in the health field ^(^ 11 ^)^ . 

 According to the WHO guidelines on digital interventions to strengthen health systems, published in 2019 ^(^ 12 ^)^ , Digital resources can facilitate communication aimed at individuals, expanding the dissemination of information quickly, complementing and improving health services. A serious game in the form of a mobile app on sexuality has great potential to complement digital health interventions. This is due to the ease with which information can be shared and disseminated. 

The objectives of this study are therefore: to develop a serious game in a mobile application for learning about safe sex and contraception with the active participation of adolescents; and to evaluate the content, appearance and usability of the technology with adolescents and experts.

## Method

### Study design and sample

This is an applied research, of technological production, on the development of a serious game, carried out in two stages: development of the technology itself; evaluation of the content, appearance and usability of the application. This research meets the recommendations of the Standards for Quality Improvement Reporting Excellence (SQUIRE 2.0), as it is an approach to improving healthcare.

 The User-Centered Design (UCD) model was adopted for the development stage ^(^ 13 ^)^ , based on Participatory Design, which involves end users in all stages of the development process. UCD is a flexible, iterative process made up of four phases: observation; design (idea generation); prototyping; and testing. These phases can occur simultaneously and repetitively as necessary, ensuring that the product design is genuinely centered on the users and that their needs are met in the most effective way possible. 

 In view of this, it was decided to use the focus group technique. It is generally recommended that the number of participants in a focus group should be between six and fifteen individuals in order to delve deeper into the topic under discussion ^(^ 14 ^)^ . As inclusion criteria, the participants in the development stage had to be adolescents aged between 15 and 17 who were regularly enrolled in secondary school during the 2015 school year. 

 For the evaluation stage, the adolescent participants had to belong to the focus group formed at the beginning of the research, as long as they were still in the age range for adolescence, between 10 and 19 years old. In order to take part as an expert evaluator, the inclusion criterion was to be a nurse from the areas of public health and/or health technology. As for the ideal number of experts, the recommendations of the Brazilian Association of Technical Standards (ABNT) were followed, based on the International Organization for Standardization (ISO) and the International Electrotechnical Commission (IEC), which, in turn, offers ISO/IEC 25062:2011, which guides a minimum sample of eight participants ^(^ 15 ^)^ . 

### Study setting

The focus group meetings for the development of the serious game were conducted in person by the main author of this study at a public school in the city of São Paulo-Brazil.The press office of the São Paulo State Department of Education brokered the analysis and approval for the research to be carried out at this institution, which, together with the Regional Teaching Board and the school unit, verified the schedule of lectures on sexuality and safe sexual practice during that period. The choice of this environment was based on the safety of the location and suitability for data collection, as well as the convenience of access for the researcher.

### Data collection

 Data collection for the development of the serious game took place between October 2015 and February 2019 through a focus group made up of adolescents. The focus group technique provides a favorable environment for discussions and sharing experiences related to a specific topic. It promotes debate among the participants, allowing for a more in-depth exploration of the topics compared to individual interviews ^(^ 14 ^)^ . 

The conversations were recorded and lasted an average of one hour each. The guiding questions used were: “How often do adolescents who use contraceptive methods use them? What is their motivation? What doubts and difficulties do they have when using these methods during sex?”.

The evaluation stage was carried out in two stages and virtually via Google Forms, a free service for creating online forms: the first took place in February 2019 with the adolescents and the other in June 2023 with the experts, after improvements had been implemented.

### Evaluation tool

 To assess the quality of the serious game, we used a checklist - the Ergolist ^(^ 16 ^)^ , a software evaluation tool - in order to list evaluation criteria relating to the content, appearance and usability of educational technology, with experts and end users. In addition, the Likert scale was used, which involves assessing the level of agreement or disagreement with something, by selecting a point on a scale with five gradations: (1 = Strongly disagree - SD; 2 = partially disagree - PD; 3 = indifferent -I; partially agree - PA; 5 = Strongly agree - SA), making it possible to identify aspects to be improved or corrected, consolidating it as an effective way of assessing the receptivity and acceptability of the serious game in question ^(^ 5 ^)^ . 

 A recent study ^(^ 5 ^)^ used the Ergolist and the Likert scale to create his own instrument to evaluate his technological product. This same instrument was adapted and used in this study. In a simple and clear manner, it sought to identify the profile of the participants, covering data such as: academic background, work institution, position held and length of experience, where applicable, and then presented evaluation criteria relating to educational aspects (relevance of the topic, objectives and texts/hypertexts), the environment’s interface (navigability, accessibility and screen design) and teaching resources (interactivity and presentation of resources). 

Both the teenagers and the experts answered the same questionnaire, which addressed issues related to content, appearance and usability, in order to compare their evaluations during the course of this study.

### Data treatment and analysis

 The data was analyzed using the thematic content analysis technique, consisting of pre-analysis, exploration of the material and treatment of the results obtained and interpretation ^(^ 17 ^)^ . To assess the quality of the serious game, we used the Content Validity Index (CVI), which evaluates the percentage of agreement with each criterion established in the evaluation instrument. Thus, it was possible to determine the relevance and/or acceptability of the item by adding up the answers with Likert 4 and 5, dividing by the total number of possible answers, making the resulting value at least 0.80 or 80% acceptable ^(^ 18 ^)^ . 

### Ethical aspects

The study was approved by the Research Ethics Committee of the institution hosting the research, under process no. 141/2015, with the Certificate of Submission for Ethical Appraisal (CAAE) number 46919315.0.0000.5393, on October 7, 2015. The following were sent: a Free and Informed Consent Form (ICF); a Free and Informed Assent Form to parents and adolescents who agreed to take part in the research; and an invitation letter to the selected nursing professionals with information about the research, along with the ICF, the link to download the application and the Evaluation Form via Google Forms.

## Results

### Development of the serious game

A total of nine teenagers, students aged between 15 and 17, were selected for convenience by the management of the educational institution chosen for the research. Recommended to ensure the fluidity of the group meetings, they were invited to take part in the focus group meetings to collect data for the development of the serious game.

 The group was made up of three boys and six girls, regardless of their sexual orientation. They were regularly attending the 1 ^st^ year of secondary school (MS) during the 2015 school year. The choice of the 1 ^st^ year of secondary school was motivated by the continuity of the project, considering that, until the conclusion of data collection in 2019, the students would theoretically maintain characteristics consistent with the profile of adolescents, which, according to the WHO, comprises the 10-19 age group ^(^ 4 ^)^ . 

First, the group was introduced to the adolescents and the researcher, and the research was explained. The reasons for choosing the topic were explained and data on early sex and teenage pregnancy was presented. One of the teenagers dropped out of the research because he considered the subject to be irrelevant, and we continued with a group made up of eight teenagers who actively participated in the process of developing the serious game.

In order to develop the proposed technology, it was essential to obtain more specific information about the participants’ learning needs in relation to safe sex and contraception. Thus, a total of twelve focus group meetings were held at the adolescents’ own school, conducted by the main author of this study.

The meetings were used to discuss the topic in question. The conversations were recorded, transcribed and analyzed to guide the development of the content (information) and structure (prototyping) of the proposed technology, according to the suggestions made by the group.

Following the phases of the UCD, in its initial phase, called Observation, the researcher must identify a specific learning problem in nursing to be studied, elucidating the motivational factors for this choice. This stage can be conducted by means of a literature review, field research, interviews, among others.

 In this study, data was collected through a focus group. The content analysis technique was used to identify the profile of the target audience, seeking to understand their language and learning needs. After transcribing the conversations, it was possible to cut them out and put them together according to their respective recording unit, i.e. categorizing them according to their central ideas (themes). The themes found were: sexual practice; contraceptive methods; pregnancy; STIs; gambling. Some of the speeches referring to the themes are transcribed in Figure 1 . 

In the Design phase (idea generation), the aim is to find possible solutions to the problem(s) identified after analyzing the data collected. To this end, the main author of this study drew up game and content scripts to guide the creation of the serious game. The game script presented an outline of what the graphics would look like on each screen of the serious game, containing examples of images, objects, avatars, scenarios, interactions, among other items, and was approved by the teenagers taking part in the research.

The screens were organized using PowerPoint slides, a program used for creating/editing and displaying graphic presentations, and then converted into a more interactive form using the Articulate Storyline software, which is a tool for building e-learning modules and online courses. It is possible to produce simulations, questionnaires and products in a dynamic and interactive way, but it is not possible to generate files in smartphone application format.

**Figure 1:** - Key expressions spoken by adolescents and their respective central ideas. São Paulo, SP, Brazil, 2015 ^(^ 19 ^)^

**Key expressions**	**Central idea**
*It’s a subject everyone talks about, something everyone does.* *She started dating and, less than a month later, she was taking it every day.*	Sexual practice
*I don’t use the pill because I forget too much.* *People, if you take medicine, you can get pregnant. So I already take injections.*	Contraceptive methods
*[Pregnancy happens] sometimes the girl isn’t guided.* *A friend at school who got pregnant and didn’t even realize it*	Pregnancy
How do you find out if you’re infected with HIV ^*^ ? *At the beginning of sex, halfway through, then I become aware.*	STIs ^†^
*It would be good to talk about pregnancy, condoms and, above all, the disease.* *How you use the methods, the chance of getting pregnant, what to do when you forget, how long you’ve been protected for.* *It would be very interesting [the game], I would play it.*	Play

* HIV = Human Immunodeficiency Virus;

† ISTs = Sexually Transmitted Infections

Together with the adolescents, we designed an outline of the scenario with the places most frequented by this age group and short, easy-to-understand content to make up the serious game, based on their information needs. Thus, the content script covered the theoretical and informative part of the serious game, in line with official documents made available by the Ministry of Health and the Brazilian Federation of Gynecology and Obstetrics Associations (FEBRASGO), which have an indispensable collection of works on the subject. The script listed the most prevalent contraceptive methods in this age group, including: the contraceptive pill, injectable hormonal contraceptives, condoms, the diaphragm, the morning-after pill and the intrauterine device (IUD).

A diagram was made of the questionnaire specially designed for the app. Depending on each answer given by the teenager, a particular contraceptive method is indicated at the end of the questions.

In the Prototyping phase, the ideas proposed in the previous phases are put into practice. Some items should already be pre-established, such as the material to be used as the serious game’s educational content, based on evidence found in the literature, and the technology’s learning objectives.

Most of the research time was spent developing prototypes and their final version. Designers and application developers were hired subject to the availability of a technical reserve from the funding agency.

 Thus, the serious game was created using Unity 3D ^®^ , a specialized software for creating games, which offers support for both 2D and 3D environments, with resources that provide immersion and flexibility to meet the specific demands of each type of game, regardless of genre ( https://unity3d.com/pt/unity ). We chose to use the 2D format to optimize the application, reducing memory consumption on mobile devices. In addition, the touchscreen game style was chosen, allowing direct manipulation by touching the surface of the screen. 

**Figure 2:**
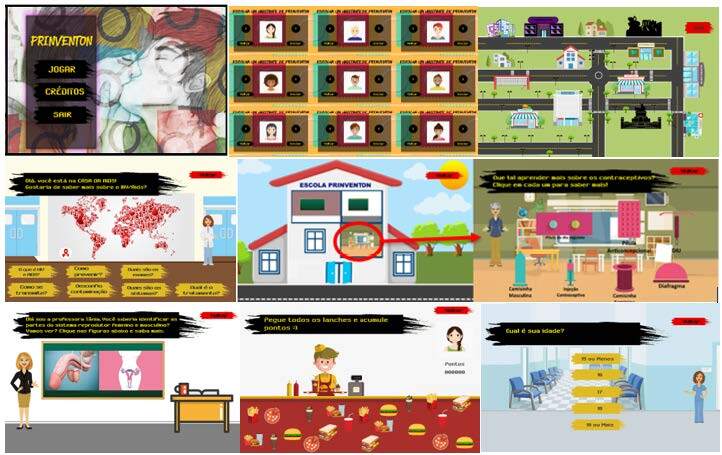
- Prinventon App ^®^ screenshots. Ribeirão Preto, SP, Brazil, 2019

 The serious game developed was called Prinventon App ^®^ ( Figure 2 ), by associating the words *Prevenção* (in Portuguese) and Prevention (in English). Prinventon App ^®^ was registered with the National Institute of Industrial Property (INPI) in 2020, process number BR 51 2021 000220-2. Smartphones or tablets running the Android operating system can download the app for free via a link or the Google Play Store, requiring 120 MB of memory for installation. 

 When the user selects “Credits”, a screen appears with information about the authors of the project, the name of the project, the research site and the funding agency that supported the development of the technology. By choosing “Play”, the user is redirected to the serious game presentation screen, where they can select their character (avatar). After choosing, the user is taken to a map of the city of Prinventon ( Figure 2 ). 

The places chosen to make up the city map were selected on the basis of the teenagers’ daily lives and according to their preferences. Some of these locations offer relaxed and more playful interactions, while others approach the content in an educational manner, but still in an engaging and entertaining way. All the objects, characters and scenarios were created for the serious game, taking into account the choices of the teenagers who took part in the project. The aim was to ensure that the content, design and usability were approached in the best possible way, involving visual aspects such as drawings, colors, fonts and texts, as well as the functionality of the buttons and the execution of the proposed activities.

Special attention was paid to the attractiveness and usability of the serious game, considering factors such as font type and size, as well as color standardization, the appearance of the buttons and the choice of hosting platform. The aim was to create a comfortable visual experience, promote ease of use and ensure quick reading. These decisions were combined with a model more suited to a young audience, adopting a more relaxed and less formal style.

In the city of Prinventon, establishments have been created for educational purposes and others for entertainment and culture. Players have the opportunity to earn and accumulate points, making the gameplay more engaging. In health institutions and schools, which offer educational activities on safe sex and contraception, it was decided not to allow points to be earned; instead, a system of immediate feedback, whether positive or negative, was implemented. This was done in order to provide players with direct reinforcement for the activity carried out.

Elsewhere in the virtual city, players have the opportunity to visit and spend their accumulated points by buying objects of interest. This approach aims to encourage players to accumulate points in order to use them, thus encouraging greater interaction with the serious game’s virtual environment, creating an additional motivating element for them to explore and become more deeply involved in it.

 Below are some examples of Prinventon App ^®^ screens ( Figure 2 ). 

The main aim of the serious game is to inform teenagers about safe sex and the use of contraceptive methods. During gameplay, the app presents information on STIs, contraceptive methods and offers guidance on the most appropriate method for each situation. There is also a questionnaire that the user answers in order to receive advice on the best contraceptive method to use according to their lifestyle and purpose for using contraception.

In addition to the interaction provided by the serious game itself, players have the opportunity to access, on one of the screens referring to the school, links to websites and blogs containing complementary information. This enriches the player’s experience, allowing them to obtain additional information and delve deeper into the topic. These external resources can provide up-to-date information, educational resources and other content related to the topic, enriching the learning and awareness experience provided by the serious game.

In the Testing phase, the last phase of the UCD, the target audience of the technology uses and evaluates it, in order to check whether there is a match between their learning needs and the educational material developed.

### Evaluation of the serious game

Once the application development phase was completed, the serious game evaluation stage followed, the last phase of the UCD.

 During the research period, there were some dropouts and loss of contact with participants, mainly due to the extended duration of the project until the product was finished and ready for evaluation. These withdrawals and losses of contact are common challenges in long-term studies and can occur due to various factors, such as changes in participants’ personal circumstances or other commitments that arise over time ^(^ 20 ^)^ . 

Thus, four adolescents took part in this stage in February 2019, belonging to the focus group set up at the beginning of the study, and eight experts in the areas of public health and health technology who carried out the evaluation in June 2023 after improvements had been implemented. To select the experts, a convenience search was carried out through professional contacts and research groups related to the areas of interest.

The items used for evaluation were associated with educational aspects such as the relevance of the topic, objectives and texts/hypertexts (items 1, 2, 3, 4, 5, 6 and 20), the environment’s interface such as navigability, accessibility and screen design (items 7, 8, 9, 10, 11, 12 and 13) and teaching resources such as interactivity and the presentation of resources (items 14, 15, 16, 17, 18 and 19).

 According to the teenagers’ evaluations, the app received 90% positive evaluations (strongly agree and partially agree), 8.75% neutral evaluations (indifferent) and 1.25% negative evaluations (partially disagree and strongly disagree) ( Table 1 ). 

**Table 1:** - Distribution of the responses of the four adolescents according to the proposed items and degree of agreement and disagreement. São Paulo, SP, Brazil, 2019

**Items**	**SD** * **1**	**PD** † **2**	**I** ‡ **3**	**PA** § **4**	**SA** || **5**
The topic is relevant to the target audience (teenagers).	0	0	0	0	4
The app addresses the topic of safe sex and contraception.	0	0	0	0	4
The text is short, clear and coherent.	0	0	0	3	1
The vocabulary corresponds to adolescent language.	0	0	0	1	3
The content is sufficient.	0	0	0	3	1
The information is current and in line with the Ministry of Health.	0	0	0	1	3
It was easy to download the app.	0	0	1	1	2
I liked the name of the game.	0	1	1	2	0
The game buttons worked perfectly.	0	0	0	0	4
The content is well organized in the game.	0	0	0	1	3
I liked the colors of the game and the font used in the text.	0	0	0	1	3
The amount of information on each screen is satisfactory.	0	0	1	1	2
I didn’t encounter any bugs or glitches while playing.	0	0	0	0	4
I found the game very interactive.	0	0	1	1	2
I found the figures, images and photos to be of good quality.	0	0	0	2	2
The game’s soundtrack is in keeping with the style of the game.	0	0	1	1	2
I was able to explore the entire game.	0	0	0	1	3
I found the game dynamic.	0	0	2	1	1
I would recommend the app to anyone.	0	0	0	3	1
I believe that adolescents can change their sexual and reproductive behavior through the influence of the game.	0	0	0	3	1

* SD = Strongly Disagree;

† PD = Partially Disagree;

‡ I = Indifferent;

§ PA = Partially Agree;

|| SA = Strongly Agree

Overall, the teenagers liked the app and didn’t have any major difficulties downloading and using it. They rated it as interactive, of good quality and with sufficient content. They also highlighted the presence of group-specific environments, such as baile funk and cultural spaces, as a more relaxed and realistic approach. They expressed a high probability of recommending the app to others in their age group, indicating that it was useful enough to share with their peers.

 As for the experts, the overall CVI was 0.80, with CVI = 0.89 for the educational aspects, CVI = 0.62 for the environment interface and CVI = 0.89 for the teaching resources ( Table 2 ). 

The experts considered the app to be very intuitive, perceiving the topic covered as relevant to technology, especially the approach to safe sex and contraception. In general, they mentioned that the vocabulary used was appropriate, using a language close to that of adolescents, and the information was up-to-date, suggesting the possibility of incorporating more content. However, some encountered difficulties when trying to install the app on their smartphones.

According to them, the objects arranged on each screen of the app are in conformity, meeting quality standards, as well as presenting attractive colors. In addition, the inclusion of a soundtrack helped to make the serious game more interactive and dynamic.

As a result, the app received 90% positive responses from adolescents and had a CVI of 0.80 from nursing experts, which is considered adequate. They rated the app as interesting and important in terms of the subject matter.

**Table 2:** - Distribution of the responses of the eight expert nurses according to the proposed items and degree of agreement and disagreement. São Paulo, SP, Brazil, 2023

**Items**	**SD** ^*^ **1**	**PD** ^†^ **2**	**I** ^‡^ **3**	**PA** ^§^ **4**	**SA** ^||^ **5**
The topic is relevant to the target audience (teenagers).	0	0	0	1	7
The app addresses the topic of safe sex and contraception.	0	0	0	3	5
The text is short, clear and coherent.	0	0	1	4	3
The vocabulary corresponds to adolescent language.	0	0	2	4	2
The content is sufficient.	0	1	1	1	5
The information is current and in line with the Ministry of Health.	0	0	0	4	4
It was easy to download the app.	0	1	3	1	3
I liked the name of the game.	0	1	4	2	1
The game buttons worked perfectly.	0	1	2	2	3
The content is well organized in the game.	0	0	2	4	2
I liked the colors of the game and the font used in the text.	0	0	1	4	3
The amount of information on each screen is satisfactory.	0	0	2	4	2
I didn’t encounter any bugs or glitches while playing.	1	2	1	1	3
I found the game very interactive.	0	0	1	3	4
I found the figures, images and photos to be of good quality.	0	0	2	5	1
The game’s soundtrack is in keeping with the style of the game.	0	1	1	3	3
I was able to explore the entire game.	0	0	0	1	7
I found the game dynamic.	0	0	0	5	3
I found the game very interactive.	0	0	0	6	2
I believe that adolescents can change their sexual and reproductive behavior through the influence of the game.	0	1	0	5	2

* SD = Strongly Disagree;

† PD = Partially Disagree;

‡ I = Indifferent;

§ PA = Partially Agree;

|| SA = Strongly Agree

It was noted that in the adolescents’ negative evaluations, including the “indifferent” criterion, one of them mentioned a slight difficulty in downloading the serious game onto their smartphone, while another pointed out that there was a greater volume of information on certain screens and that it would be possible to reduce the amount of text. The experts, meanwhile, recommended changes to the design of the screens, such as the background color of the text frames which seemed dark and improving the framing, as well as correcting spelling and grammar. Another aspect that was checked was the amount of text on each screen, as two evaluators highlighted the presence of excessive information on some of them. They pointed out the need to change corrupted links and insert guidelines on how to play, such as a tutorial, which was not included in the serious game.

The suggested reformulations were incorporated with the aim of improving the accessibility of the technology for teenagers.

## Discussion

During data collection for the development of the serious game, it was noted that there are uncertainties regarding the use of contraceptives, their effectiveness, benefits and side effects. There was considerable emphasis on preventing unintended pregnancy and a slight concern about STIs. Notably, there was no immediate mention of condoms for practicing safe sex.

The speeches transcribed for this study reinforce the need for an intervention that is effective in building knowledge about safe sex and contraception. In this sense, the topics covered in the serious game aimed to address the doubts raised, using content derived from the available literature as a basis. There was also discussion about how the topics in question should be approached in the technology and how the information would be presented, seeking to meet the expectations of the adolescents and the technical reserve available to spend during the creation of the product.

 The importance of a user-friendly interface should be considered in order to achieve a positive impact on the teaching-learning process through the use of educational technologies ^(^ 20 ^)^ . Therefore, when designing the serious game, priority was given to creating a virtual city in which the player could explore and interact with the available locations, all based on their social context. 

 Usability in games is also paramount, since its absence can result in user frustration, leading to reduced performance and motivation. Therefore, addressing usability in games is a highlight, since users value games that are intuitive and engaging ^(^ 21 ^)^ . 

 In developing this app, we sought originality, especially in the context of sexual and reproductive health education, with an emphasis on safe sex and contraception. This was done by considering aspects covered in four studies related to the topic in question, two from international literature and two from national literature. Two Chinese studies developed serious games for adolescent sex education with a focus on contraception: Making Smart Choices ^(^ 22 ^)^ and UnderControl ^(^ 23 ^)^ . The first had a moderate impact on users, promoting positive attitudes towards sex and encouraging more assertive and safer sexual decision-making, considering the country’s socio-cultural scenario. In the second study, the playability of UnderControl was evaluated based on user satisfaction with the learning process. 

 The other two games, of Brazilian origin and centered on the theme, are: DECIDIX ^(^ 24 ^)^ , whose aim is to encourage reflection on the affective and sexual relationships that can culminate (or not) in an unintended pregnancy. This takes place through a chat in which the participants collectively discuss and choose the directions the characters should take in the story; and “Stay Alert” ^(^ 25 ^)^ , which aims to disseminate knowledge about sexuality and contraceptive methods through quizzes, sharing, tips and messages. 

The aforementioned studies highlighted the importance of improving the games developed, emphasizing the creation of tutorials to guide users on how to play and the development of scenarios that are more in line with the reality of adolescents, taking into account the various socio-cultural contexts. In this context, the serious game resulting from this study represents a step forward as it was developed in a participatory way with adolescents, rather than just for them.

In addition, as the serious game is set in a virtual city in which teenagers feel integrated, actively participating in the process due to the level of immersion and interaction, it offers scenarios that reflect their daily lives. This includes emblematic locations such as funk clubs, health units, schools and historical monuments specific to the city of São Paulo, where the participatory development of the serious game took place.

The language incorporated into the app was designed specifically for the teenage target audience. Young people have a unique way of expressing themselves, with a current language that differs from previous generations, including slang and popular expressions. The choice of informal language in the app aims to bring teenagers closer to the experience, making it more understandable and motivating for this specific group. Each object, character and scenario present in the serious game was developed with the aim of meeting the demands of technology, with the collaboration of designers.

To further enrich the user experience, the serious game includes, on certain screens, links to blogs and websites developed by the Ministry of Health and other institutions. All the teenagers who took part in the survey expressed the habit of looking for information on contraception and sexual practices on the internet, as well as receiving information from friends who have already had sexual experiences. Therefore, the inclusion of additional sources of consultation is fundamental to improving the user experience by providing reliable and up-to-date information related to sexual and reproductive health.

These external resources not only complement the learning provided by the serious game, but also contribute to increasing adolescents’ awareness of the topic under discussion.

It is understandable that the development of the technology was conducted with limited resources from a funding agency, considering the challenges faced by science in the country throughout the research. This reality influenced the need to restrict the complexity of the application in order to optimize the available resources.

 As a result, the Prinventon App ^®^ was completed in its development, taking into account the choices made by the teenagers themselves and the financial resources available at the time. Improvements were implemented in response to the evaluators’ suggestions. After a comprehensive evaluation, it was found that the serious game is an important resource to be used by adolescents, and is recognized as a tool with great potential to benefit the health of this population. 

 The technologies used for health education, in the case of this study, the use of serious games, make a significant contribution to knowledge, while at the same time providing entertainment. When aimed at adolescents, they have had a positive influence on their educational process, as they take an active role in their own development and learning ^(^ 26 ^-^ 27 ^)^ . Thus, the app is considered important for the health field and the results of this research should be disseminated to contribute to future research related to the advancement and use of technologies aimed at long-term health education. 

 The WHO highlights the relevance of more detailed evaluations of the impacts of digital interventions, as these analyses can provide important information for understanding the effects of the resulting changes on users over time ^(^ 12 ^)^ . Therefore, long-term research would be essential to analyze and validate the potential impact of Prinventon App ^®^ on contraceptive knowledge and related behavioral changes among adolescents. 

In order to ascertain whether the technology developed has an effect on knowledge and use of contraceptive methods, further research needs to be conducted. This should be conducted by means of an experimental study, in order to compare contraceptive practices aimed at safe sex before and after using the app and measuring knowledge on the subject, covering a larger sample of adolescents.

Thus, this type of research requires a new study to continue an analysis that would allow the progress of adolescents using the app to be monitored over time, making it possible to assess improvements in knowledge about contraception and positive changes in their behaviors related to sexual health. In addition, it would be possible to identify which aspects of the app had the greatest impact and which areas needed adjustment or improvement.

 Furthermore, longitudinal studies to assess the long-term impact of Prinventon App ^®^ would be an important approach to better understand how this digital intervention is contributing to adolescents’ education and awareness of sexual health and contraception. 

It is anticipated that nurses will maintain a continuously updated professional profile, adapting to technological innovations in the field of health education. This improvement focuses on user care and seeks technological training to address challenges encountered in professional practice, either through direct experience or through evidence from specialized literature. These skills and competencies are essential for nursing practice, with a view to promoting health and ensuring patient safety.

## Conclusion

 The process of developing the Prinventon App ^®^ serious game on safe sex and contraception involved direct collaboration with adolescents, ensuring that their choices were incorporated at every stage. This method provided opportunities to approach the topic in an attractive and interactive way, positively adapting to the target audience. 

It should be noted that the topics covered in the app, such as sexual practice, contraceptive methods, pregnancy and STIs, involve an abundance of significant information. Therefore, selecting the most relevant and essential content was fundamental to ensuring the app’s effectiveness in educating and raising awareness among teenagers about the subject.

It is essential to consider strategies that allow the active use of the serious game in a contextualized way in the lives of adolescents, considering the contemporary socio-cultural scenario. The aim is to stimulate meaningful learning, promote changes in sexual behavior and contribute to the personal maturation of this audience.

Although there are a variety of educational materials and technologies available on the subject, in formats other than serious games, there are still areas that need to be filled in or complemented. Sexuality itself is definitely a broad topic and it was not possible to cover all its aspects in a single digital resource. Many materials and technologies complement each other and aim to provide information to users.

However, it is important to go beyond merely providing information and look for strategies that involve adolescents in an active, motivating way and that are contextualized to their reality. This can include interactive, participatory and engaging approaches, such as the use of educational games, to promote more meaningful learning and promote positive behavioral changes related to sexual and reproductive health.

 Overall, the Prinventon App ^®^ met the expectations of adolescents and nursing professionals, although it needs some implementation. It is suggested that future research verify its effectiveness in improving adolescents’ knowledge and changing their contraceptive behavior. In order to do this, a new long-term study needs to be carried out, covering a larger number of users of the app. 
